# A Rare Case of Disseminated Granular Cell Tumors Throughout the Gastrointestinal Tract Diagnosed by Endoscopic Ultrasonography Associated With Endoscopic Mucosal Resection

**DOI:** 10.7759/cureus.107909

**Published:** 2026-04-28

**Authors:** Lara Kodra, Amanda A Lage, Marcella Wanderley, Carolina T Pinheiro, Pedro B de Oliveira, Fernanda C Barroso, Filadélfio E Venco, Jose C Ardengh, Ana M Zuccaro

**Affiliations:** 1 Endoscopy, Hospital Federal de Ipanema, Rio de Janeiro, BRA; 2 Gastroenterology and Endoscopy, Hospital Federal de Ipanema, Rio de Janeiro, BRA; 3 Pathology, Fila Medicina e Patologia, São Paulo, BRA; 4 Gastrointestinal Endoscopy, Hospital das Clínicas de Ribeirão Preto, Ribeirão Preto, BRA; 5 Imaging Diagnostics, Universidade Federal de Sao Paulo, São Paulo, BRA

**Keywords:** abrikossoff tumor, diagnosis of rare cases, endoscopic advanced treatment, endoscopic mucosal resection, endoscopic ultrasound (eus)

## Abstract

Granular cell tumor (GCT) is a neoplasm of uncertain histopathological origin; therefore, no uniformly accepted treatment strategy has yet been established. Microscopically, it is characterized by eosinophilic granules. This tumor involves the skin and subcutaneous tissue. Gastrointestinal involvement is uncommon; however, isolated granular cell tumors of the esophagus, stomach, and colon, as well as multifocal lesions of the esophagus and stomach, have been reported. Diffuse involvement of the entire gastrointestinal tract has not been described.

We report the case of a 20-year-old woman who presented with a chronic cough. Chest computed tomography revealed a retrotracheal mass, which was surgically resected and ultimately diagnosed as a GCT. Upper gastrointestinal endoscopy, performed to investigate dyspepsia, revealed multiple synchronous GCTs throughout the esophagus, stomach, and duodenum. Endoscopic ultrasound of the largest identified nodule revealed a well-defined hypoechoic lesion confined to the second hypoechoic layer; all remaining lesions demonstrated similar ultrasonographic features. This lesion was resected "in bloc" without adverse events using endoscopic mucosal resection for diagnostic and therapeutic purposes.

Immunohistochemical (IHC) analysis demonstrated positivity for S100 and CD68, with expression of CD34 also observed. The patient has been followed on an outpatient basis, and symptoms of postprandial fullness and abdominal discomfort have been managed conservatively, with satisfactory symptomatic relief to date.

Immunohistochemistry was sufficient for the definitive classification of the lesion as a GCT, and endoscopic mucosal resection appears to be an appropriate diagnostic approach for lesions larger than 20 mm. Furthermore, the authors discuss the optimal surveillance strategy for patients with disseminated GCTs of the gastrointestinal tract.

## Introduction

Abrikossoff described the granular cell tumor (GCT) in 1926 [1-3]. It is a rare neoplasm of soft tissue whose histogenesis remains controversial; however, current evidence suggests that GCTs arise from Schwann cell differentiation [1-3]. In a series of 410,000 surgical specimens, the incidence of GCTs was 0.03% [4]. GCTs may develop in virtually any anatomical site, although they most commonly involve the skin and subcutaneous tissues of the head, neck, trunk, extremities, and vulva. Gastrointestinal tract involvement accounts for only 6% of all GCTs [5]. Within the gastrointestinal tract, GCTs predominantly affect the esophagus and colon, whereas gastric involvement is particularly uncommon, and disseminated disease affecting all segments of the digestive system is exceedingly rare [5].

An et al. reported a series of 98 gastrointestinal GCTs, comprising 73 esophageal (75%), 21 colorectal (21%), and 4 gastric (4%) cases [5]. The rate of malignant transformation has been estimated at approximately 2% [5-7]. Gastric GCT (GGCT) is typically detected by upper gastrointestinal endoscopy (UGE) and endoscopic ultrasound (EUS). On UGE, GCTs appear as small, sessile, yellowish-white lesions covered by intact overlying mucosa [7]. Histologically, the tumor is characterized by eosinophilic cytoplasmic granules, and immunohistochemical (IHC) analysis demonstrates positivity for S-100 protein, CD68, and the transcription factor SOX-10 [8].

Like most subepithelial tumors (SETs), GCTs arise from the deeper layers of the gastrointestinal wall - such as the submucosa or muscularis propria - and are invariably covered by normal-appearing mucosa, imparting a bulging or elevated appearance on endoscopy. This feature poses a diagnostic challenge when conventional biopsy techniques are employed, as the intact mucosal surface frequently precludes adequate tissue sampling [9]. EUS, which integrates endoscopy and ultrasonography within a single instrument, enables comprehensive characterization of SETs, including assessment of location, size, echogenicity, and margins, thereby facilitating both diagnosis and therapeutic planning [10].

Although GCTs are benign in the majority of cases, they occasionally exhibit aggressive behavior, including local recurrence or distant metastasis [11,12]. The optimal treatment strategy remains debated, with available options including endoscopic mucosal resection (EMR), endoscopic submucosal dissection (ESD), and conventional surgical resection [6,7].

The present study reports a patient with GCTs disseminated throughout the mediastinum, esophagus, stomach, small intestine, and colon. Only the largest identified lesion was resected under EUS guidance; subsequent histopathological and immunohistochemical analysis confirmed the diagnosis of GCT. The objective of this study was to contribute to the diagnostic workup and to expand current understanding of the diagnostic and therapeutic approaches applicable to multifocal and/or disseminated GCTs.

## Case presentation

A 20-year-old woman presented with a two-year history of cough. Diagnostic workup identified a retrotracheal mass, which was subsequently resected. Histopathological examination confirmed the diagnosis of mediastinal GCT. Several months later, the patient developed right upper quadrant pain radiating to the back, triggered by fatty meal ingestion.

**Figure 1:**
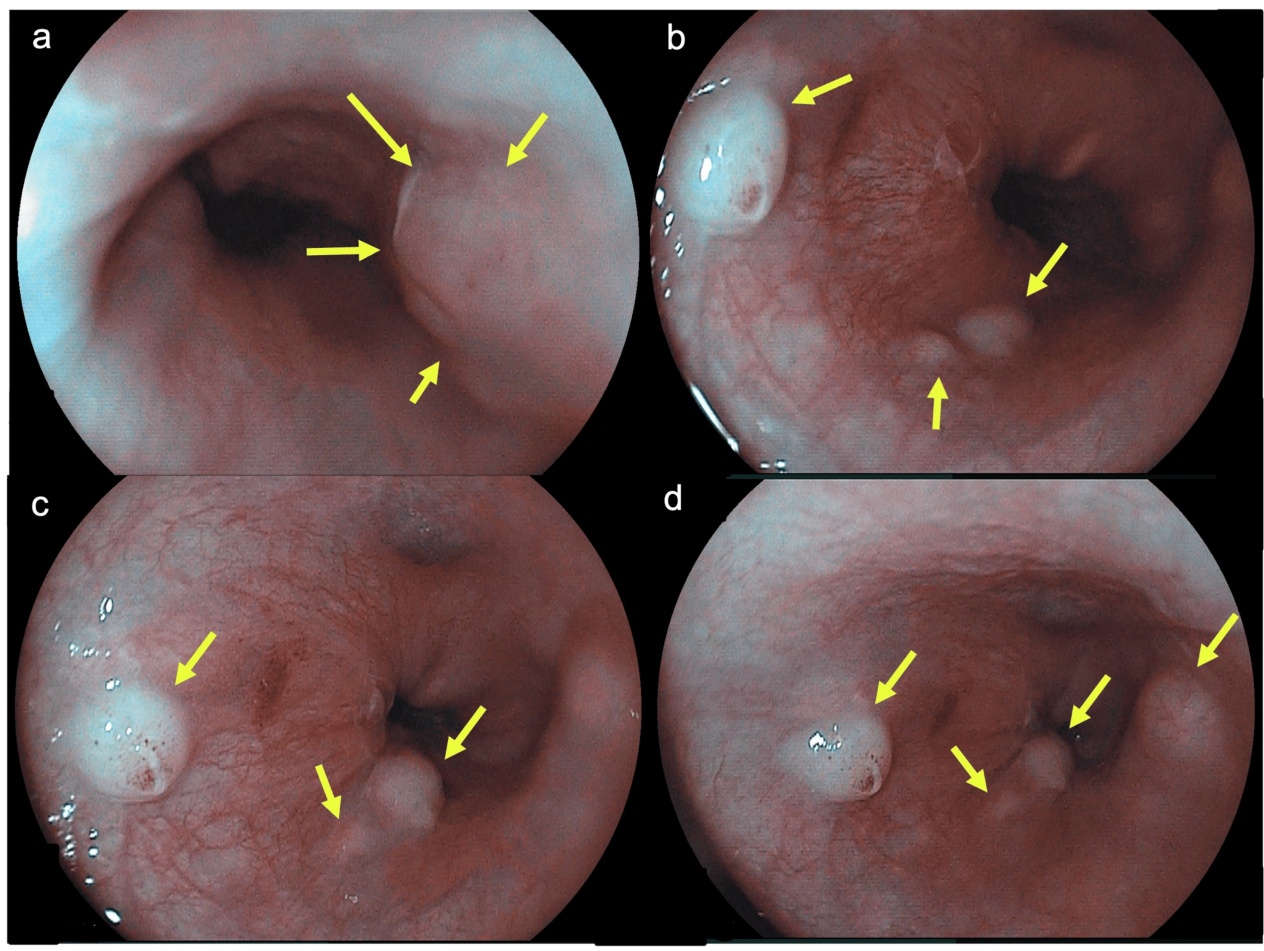
(a) Endoscopic image of the proximal esophagus showing a 15 mm SET (yellow arrows). (b), (c), and (d) Endoscopic images of the distal esophagus showing multiple SETs of approximately 10 mm distributed along all esophageal walls. SET: subepithelial tumor

She denied comorbidities, known drug allergies, and alcohol or tobacco use. Physical and dermatological examinations were unremarkable. Abdominal ultrasound revealed cholelithiasis, and upper gastrointestinal endoscopy (UGE) demonstrated multiple SETs distributed multifocally throughout the middle and distal esophagus. The lesions exhibited a fibroelastic consistency, were covered by intact yellowish-white mucosa, and measured between 3 and 6 mm (Figure 1).

**Figure 2:**
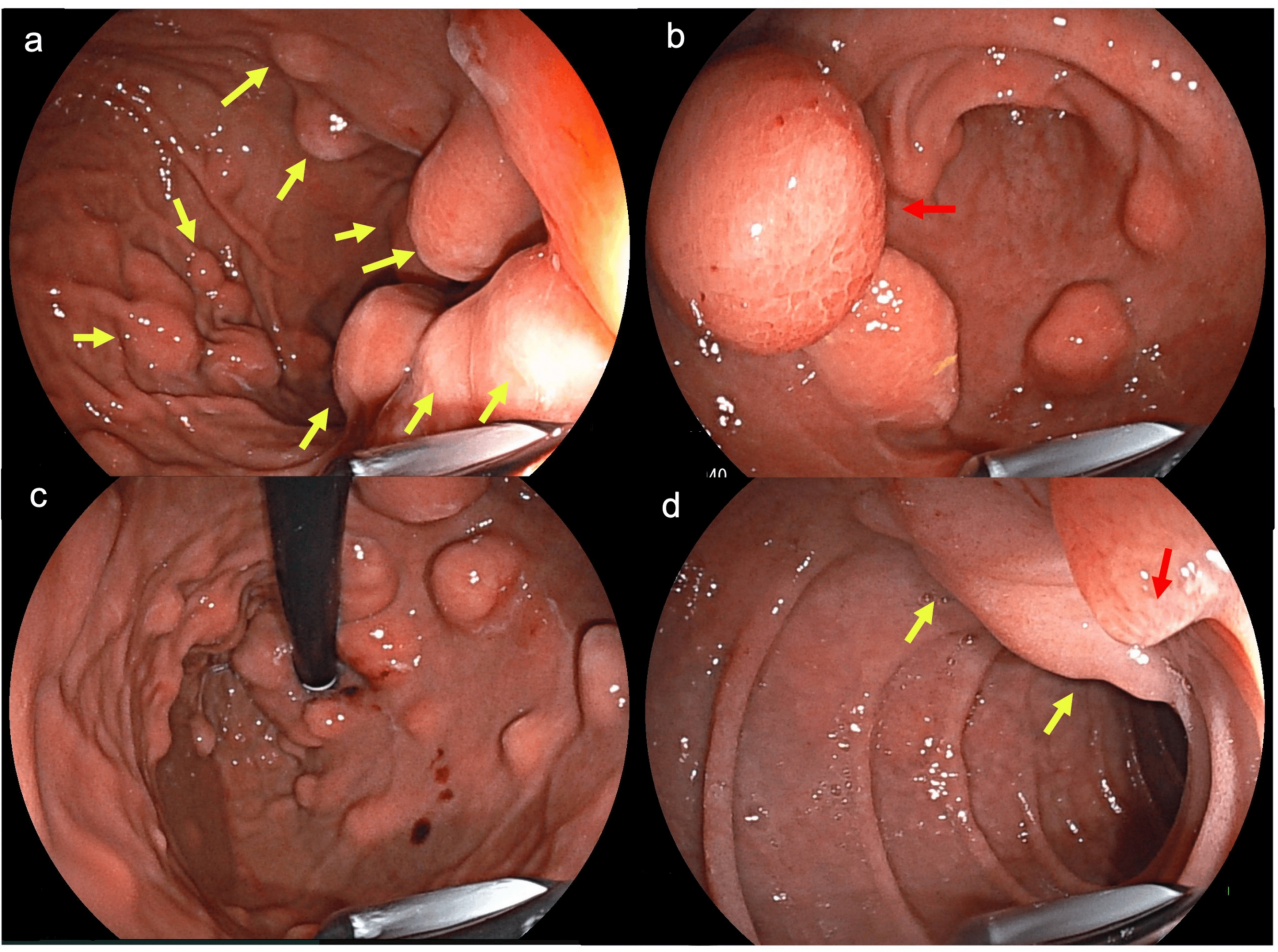
(a) Multiple SETs of varying sizes in the gastric body (yellow arrows). (b) SETs at the gastric body–antrum junction; the largest (>20 mm) is located on the anterior wall (red arrow). (c) Similar lesions in the gastric fundus. (d) SET in the duodenum, adjacent to the major duodenal papilla (yellow and red arrows). SET: subepithelial tumor

Additional SETs with similar endoscopic features were identified throughout the stomach - including the fundus, body, and antrum - with the largest measuring 20 mm. Laboratory investigations were unremarkable. One year later, EUS demonstrated multiple hypoechoic, ovoid SETs of varying sizes, confined to the second endosonographic layer and distributed throughout the esophagus, stomach, and duodenum (Figure 2).

**Figure 3:**
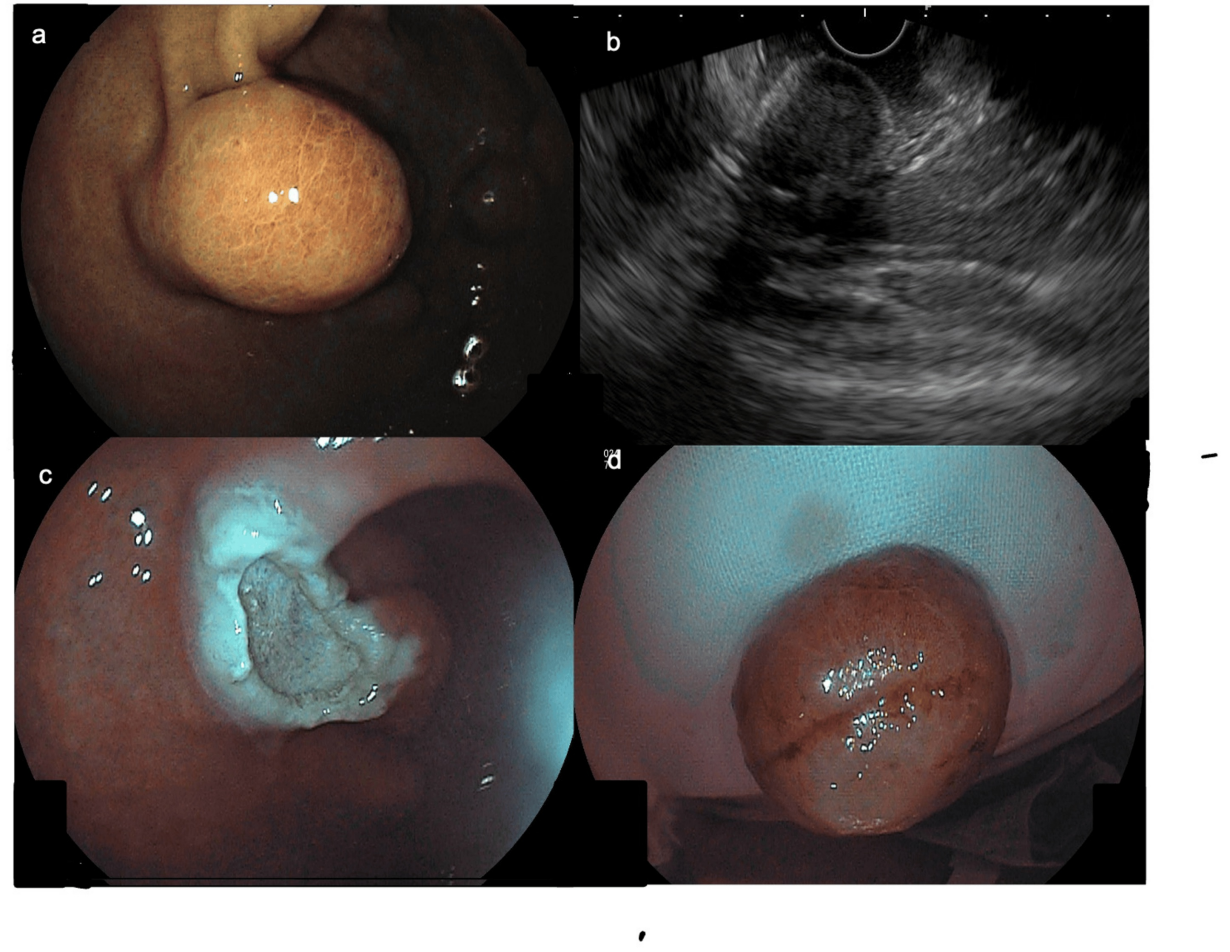
(a) Endoscopic view of the largest SET. (b) EUS showing an ovoid, homogeneous, well-demarcated 20 mm lesion arising from the second endosonographic layer. (c) Mucosal defect after EMR. (d) Resected specimen. SET: subepithelial tumor; EUS: endoscopic ultrasonography; EMR: endoscopic mucosal resection

The largest lesion, measuring 20 mm and located on the anterior wall of the gastric antrum, was resected using the EMR technique (Figure 3).

**Figure 4:**
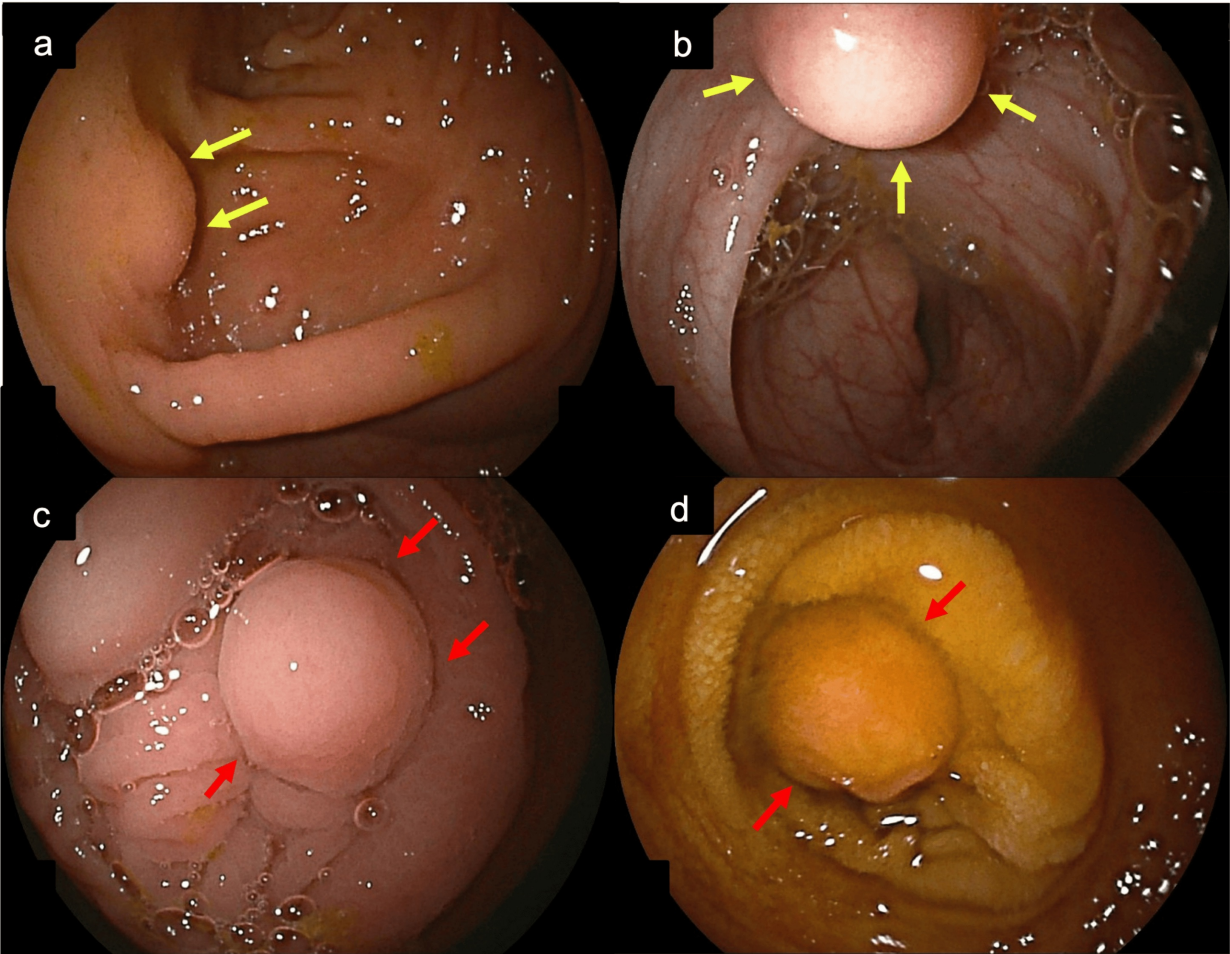
(a) SET in the transverse colon with features similar to those of lesions identified in other gastrointestinal segments (yellow arrows). (b) Additional colonic SET with features identical to the previously described lesions (yellow arrows). (c) and (d) SETs in the terminal ileum consistent with all previously described lesions (red arrows). SET: subepithelial tumor

Given the multifocal and disseminated distribution of SETs involving the mediastinum and upper gastrointestinal tract, colonoscopy with ileoscopy - advancing more than 50 cm beyond the ileocecal valve - was performed. SETs measuring 5-12 mm were identified across all colonic segments, with an endoscopic appearance consistent with that of the upper gastrointestinal lesions. Additional lesions presenting as mildly polypoid, whitish-brown nodules were also identified in the terminal ileum (Figure 4).

**Figure 5:**
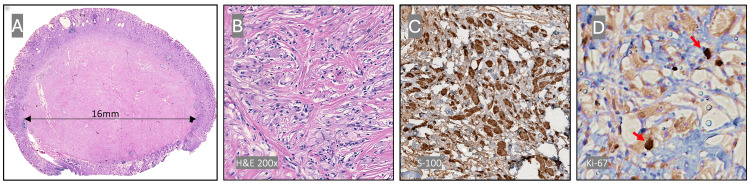
(a) Mucosectomy specimen showing a well-defined, completely resected neoplasm. (b) Histology showing abundant eosinophilic granular cytoplasm, rounded nuclei, and no cellular atypia. (c) IHC: S-100 strongly and diffusely positive. (d) Ki-67: 1%.

Histopathological examination (H&E) complemented by immunohistochemical (IHC) analysis confirmed the diagnosis of GCT. The lesion consisted of a non-encapsulated, well-demarcated nodule adherent to the muscularis mucosae, composed of polygonal to spindle-shaped cells with abundant, finely granular eosinophilic cytoplasm (phagolysosome aggregates). The nuclei were small and bland, with no mitotic activity. The stroma was scant and mildly collagenized, and necrosis was absent. Immunohistochemistry demonstrated strong and diffuse positivity for S-100, consistent with neuroectodermal differentiation (Figure 5). The Ki-67 proliferation index was less than 1%, reflecting the benign nature of the lesion; malignant transformation occurs in approximately 0.5-2% of cases.

## Discussion

The histology of GCTs reveals tumor cells whose morphology resembles that of skeletal muscle cells, which led to their initial designation as "granular cell myoblastomas." Positivity for S-100 protein, together with the granular lysosomal ultrastructure, supports differentiation toward Schwann cells [1,2]. In the present case, the resected GGCT expressed CD34; however, this expression was not observed in the tumor cells themselves. Rather, immunohistochemical analysis demonstrated CD34-positive pericellular matrix surrounding tumor cells arranged in nests, further supporting the hypothesis of Schwann cell origin. The most recent literature review identified 42 documented cases of GGCTs, the majority of which are solitary; however, these tumors may also present as multiple lesions at different anatomical sites within the same individual [4] or involve multiple organs simultaneously [4,13-18].

GCTs most commonly involve the esophagus or colon. With regard to the topographic distribution of GGCTs, no clear site predilection has been identified, as lesions may develop in the gastric cardia, body, or antrum. The mean age at presentation is 48 years (median 50 years; range 16-76 years) [4], and no significant predisposition based on sex or race has been documented [4]. As reported by Patti et al. [1], GGCTs are most commonly identified in patients aged 40 to 60 years with no clear sex predilection - a pattern that contrasts with the present case, in which the patient was first diagnosed with a mediastinal GCT at 19 years of age. Available evidence suggests that individuals of African and Japanese descent may be disproportionately affected [4].

Patients with GGCTs often present with postprandial fullness, abdominal discomfort, pain, or dyspepsia, and these tumors are frequently detected incidentally during imaging, endoscopy, or unrelated surgical procedures. In the present case, a 20-year-old patient with nonspecific gastrointestinal complaints was found to have GCTs involving multiple sites throughout the upper gastrointestinal tract. When ulcerated, GGCTs may cause gastrointestinal bleeding. Rare cases of concomitant occurrence with gastric adenocarcinoma, esophageal carcinoma, and gastric ulcers have also been reported [4,7].

Macroscopically, GGCTs present as SETs covered by yellowish, yellowish-white, or apparently normal mucosa, resembling polypoid, hemispheric, and predominantly sessile protrusions. They are firm, well-demarcated nodules that may occasionally involve the muscularis propria or serosa [7,11]. To date, 48 GGCTs have been reported in 42 patients. The typical lesion is small, with 79% of cases measuring less than 2 cm; the largest documented specimen measured 7 cm [13] - broadly comparable to the present case, in which the majority of identified GCTs measured less than 20 mm.

EUS enables assessment of location, size, echotexture, and margins of SETs, facilitating differential diagnosis and evaluation of endoscopic resectability [10]. Computed tomography may reveal well-circumscribed intramural nodules with little to no contrast enhancement, while also permitting evaluation of adjacent organs and regional lymph nodes [9]. In the present case, EUS demonstrated a well-defined hypoechoic lesion exceeding 20 mm, confined to the second sonographic layer, supporting the suitability of endoscopic removal via EMR.

Differentiation of GCTs from other SETs based on imaging alone is challenging, and definitive diagnosis requires histopathological confirmation. EUS-guided fine-needle biopsy (EUS-FNB) can yield adequate diagnostic tissue [9]; however, given that the largest identified lesion (>20 mm) was submucosal, covered by grossly normal mucosa, and confined to the superficial gastric wall, EMR was selected as the preferred diagnostic and therapeutic approach.

GCTs arising at any segment of the digestive tract share identical histological features, consisting of large polygonal or ovoid cells with abundant eosinophilic cytoplasmic granules [5,7]. In a study of 98 cases, immunohistochemistry demonstrated positivity for S-100 (100%), CD56 (100%), SOX10 (100%), CD68 (67%), and inhibin-α (33%) [5]. In the present case, the tumor stained positive for S-100, CD68, and SOX10, with negative results for inhibin-α, consistent with patterns described in the literature.

Although approximately 98% of GGCTs are benign, cases of malignant behavior with local recurrence have been documented; distant metastasis remains the only definitive criterion for malignancy [13]. A key diagnostic consideration involves the six histological criteria outlined by Fanburg-Smith et al. for differentiating benign from malignant GCTs [19]: (i) presence of necrosis; (ii) spindle-shaped cell morphology; (iii) nuclear pleomorphism; (iv) conspicuous vesicular nucleoli; (v) an elevated nuclear-to-cytoplasmic ratio; and (vi) heightened mitotic activity (>2 mitoses per 10 high-power fields at 200× magnification) [19]. Tumors meeting three or more of these criteria are classified as malignant, whereas those meeting only one or two are considered atypical [19]. None of these features was identified in the present patient; however, given the disseminated distribution of disease throughout the gastrointestinal tract, rigorous endoscopic surveillance remains imperative.

GCTs rarely metastasize, and when metastasis occurs, it typically results from local recurrence secondary to incomplete resection [13]. Although the patient's symptoms have not fully resolved, establishing a definitive diagnosis has contributed meaningfully to clinical management by providing reassurance and improving adherence to follow-up.

No established consensus exists on the optimal management of GGCTs. In the largest available review, comprising 42 patients, 30 underwent surgical treatment - including partial gastrectomy, wedge resection, or local excision [4]. Total gastrectomy was performed in three cases due to suspected lymph node involvement, multiple GGCTs, or associated adenocarcinoma. Endoscopic resection was used in nine patients, and aside from a single malignant GGCT that recurred after two years, no recurrence or metastasis was reported during follow-up [4].

These data raise the question of whether a similar management approach should be adopted for the present patient, who has multiple GCTs distributed across all segments of the gastrointestinal tract. The 2022 European Society of Gastrointestinal Endoscopy (ESGE) guidelines [10] state that asymptomatic GCTs with a confirmed diagnosis do not require routine endoscopic monitoring. For symptomatic lesions or those with an uncertain diagnosis, endoscopic reassessment is advised at 3 to 6 months, followed by surveillance every 2 to 3 years for lesions smaller than 10 mm and every 1 to 2 years for lesions measuring 10 to 20 mm. For lesions exceeding 20 mm, ESGE recommends EUS follow-up at 6 months and annually thereafter [10].

Based on these parameters, the proposed surveillance strategy for the present patient consists of UGE and lower gastrointestinal endoscopy every two years, given that most identified lesions measure between 10 and 20 mm. For any lesion exceeding 20 mm, the preferred approach will be EMR preceded by EUS evaluation. The various endoscopic resection modalities have not demonstrated statistically significant differences in therapeutic outcomes.

EMR was originally introduced into clinical practice by Tada et al. in 1984 [20] through the "strip biopsy" technique, from which ESD subsequently evolved as a technically more advanced variant [10,20]. In the largest published review of 42 GGCT cases, 77% of lesions measured 2 cm or less, and no recurrence was observed among the nine patients who underwent endoscopic resection [4].

The overwhelming majority of published studies describe GCTs involving the esophagus, stomach, or colon in isolation; simultaneous multifocal involvement of both the esophagus and stomach is uncommon [15,16,18]. The pattern of disseminated involvement throughout the entire gastrointestinal tract documented in the present case is unprecedented and, given its exceptional rarity and clinical implications, fully justifies reporting.

## Conclusions

This case represents the first documented instance of GCT disseminated throughout the entire gastrointestinal tract. EUS proved indispensable for the morphological characterization of subepithelial lesions and guided the therapeutic decision to perform en bloc EMR of the largest gastric lesion. Histopathological and immunohistochemical confirmation enabled safe long-term follow-up, with systematic endoscopic surveillance established as the cornerstone of management. The proposed strategy consists of upper and lower gastrointestinal endoscopy every two years, with EMR preceded by EUS for any lesion exceeding 20 mm. Although none of the Fanburg-Smith malignancy criteria were identified, the unprecedented disseminated distribution warrants rigorous individualized surveillance and contributes to a broader understanding of the biological behavior and natural history of disseminated gastrointestinal GCTs.

## References

[1] Patti R, Almasio PL, Di Vita G (2006). Granular cell tumor of stomach: a case report and review of literature. World J Gastroenterol.

[2] Santos C, Araújo AV, Contente H, Branco C (2019). Gastric granular cell tumour, a rare entity. BMJ Case Rep.

[3] Jain A, Karegar M, Joshi A, Rojekar A (2018). Granular cell tumour in stomach: a case report. Indian J Surg Oncol.

[4] Li H, Zhang M, Zheng Y, Zhang H (2024). Gastric granular cell tumor: A case report and literature review. Oncol Lett.

[5] An S, Jang J, Min K (2015). Granular cell tumor of the gastrointestinal tract: histologic and immunohistochemical analysis of 98 cases. Hum Pathol.

[6] Watanabe Y, Watanabe M, Suehara N (2018). Early gastric cancer with diffuse heterotopic gastric glands and granular cell tumors mimicking advanced gastric cancer. Int J Surg Case Rep.

[7] Barakat M, Kar AA, Pourshahid S, Ainechi S, Lee HJ, Othman M, Tadros M (2018). Gastrointestinal and biliary granular cell tumor: diagnosis and management. Ann Gastroenterol.

[8] Kim DJ, Kim HW, Park SB (2015). A case of gastric granular cell tumor: review of literature and features of endoscopic ultrasonography. Korean J Helicobacter Up Gastrointest Res.

[9] Yasuda A, Yasuda T, Imamoto H (2020). A case of a gastric granular cell tumor preoperatively diagnosed and successfully treated by single-incision laparoscopic surgery. Surg Case Rep.

[10] Deprez PH, Moons LM, OʼToole D (2022). Endoscopic management of subepithelial lesions including neuroendocrine neoplasms: European Society of Gastrointestinal Endoscopy (ESGE) Guideline. Endoscopy.

[11] Goodman MD, Cooper PH (1972). Granular cell tumor (myoblastoma) of the stomach. A case report with ultrastructural findings and review the literature. Am J Dig Dis.

[12] Abdelwahab IF, Klein MJ (1983). Granular cell tumor of the stomach: a case report and review of the literature. Am J Gastroenterol.

[13] Taban SM, Barna RA, Dema AL, Ratiu IM, Popa O, Plopeanu AD (2021). Unexpected diagnosis for a gastric polyp: Granular cell tumor: Case report and review of the literature. Exp Ther Med.

[14] Takaya H, Kawaratani H, Kaneko M (2017). Gastric granular cell tumor in a youth excised by endoscopic submucosal dissection : A case report and literature review. Acta Gastroenterol Belg.

[15] David O, Jakate S (1999). Multifocal granular cell tumor of the esophagus and proximal stomach with infiltrative pattern: a case report and review of the literature. Arch Pathol Lab Med.

[16] Blandamura S, Altavilla G, Castoro C, Antonini C, Piazza M (1993). Combined granular cell tumour of the oesophagus and stomach: a case report and review of the literature. Ital J Gastroenterol.

[17] Saleh H, El-Fakharany M, Frankle M (2009). Multiple synchronous granular cell tumors involving the colon, appendix and mesentery: a case report and review of the literature. J Gastrointestin Liver Dis.

[18] Sigon R, Fusaro L, Monica F, Campigotto M (2024). Gastrointestinal granular cell tumor: the first report of a multifocal and potentially hereditary case. Gastroenterol Insights.

[19] Fanburg-Smith JC, Meis-Kindblom JM, Fante R, Kindblom LG (1998). Malignant granular cell tumor of soft tissue: diagnostic criteria and clinicopathologic correlation. Am J Surg Pathol.

[20] Tada M, Shimada M, Murakami F, Mizumachi M, Arima K, Yanai H (1984). Development of strip-off biopsy [article in Japanese]. Gastroenterol Endosc.

